# Immune Fitness and the Psychosocial and Health Consequences of the COVID-19 Pandemic Lockdown in The Netherlands: Methodology and Design of the CLOFIT Study

**DOI:** 10.3390/ejihpe11010016

**Published:** 2021-02-20

**Authors:** Pantea Kiani, Agnese Merlo, Hama M. Saeed, Sarah Benson, Gillian Bruce, Rosalie Hoorn, Aletta D. Kraneveld, Aurora J. A. E. van de Loo, Noortje R. Severeijns, Annabel S. M. Sips, Andrew Scholey, Johan Garssen, Joris C. Verster

**Affiliations:** 1 Division of Pharmacology, Utrecht Institute for Pharmaceutical Sciences, Utrecht University, 3584CG Utrecht, The Netherlands; p.kiani@uu.nl (P.K.); a.merlo@uu.nl (A.M.); m.saeed@uu.nl (H.M.S.); rosaliehoorn@gmail.com (R.H.); a.d.kraneveld@uu.nl (A.D.K.); a.j.a.e.vandeloo@uu.nl (A.J.A.E.v.d.L.); n.r.severeijns@students.uu.nl (N.R.S.); a.s.m.sips@students.uu.nl (A.S.M.S.); j.garssen@uu.nl (J.G.); 2 Centre for Human Psychopharmacology, Swinburne University, Melbourne, VIC 3122, Australia; sarahmichellebenson@gmail.com (S.B.); andrew@scholeylab.com (A.S.); 3 Division of Psychology and Social Work, School of Education and Social Sciences, University of the West of Scotland, Paisley PA1 2BE, UK; gillian.bruce@uws.ac.uk; 4 Clinical Psychology, Faculty of Behavioural and Movement Sciences, Vrije Universiteit Amsterdam, 1081BT Amsterdam, The Netherlands; 5 Global Centre of Excellence Immunology, Nutricia Danone Research, 3584CT Utrecht, The Netherlands

**Keywords:** COVID-19, lockdown, immune fitness, mood, alcohol consumption, pain

## Abstract

This article provides an overview of the design and methodology of the “Corona lockdown: how fit are you?” (CLOFIT) study, including the questionnaires and scales that were included in the online survey. The aim of the CLOFIT study was to investigate the psychosocial and health consequences of the coronavirus disease 2019 (COVID-19) pandemic in the Netherlands. The survey was conducted among the Dutch population to collect data on immune fitness and the psychological and health consequences of the 2019 coronavirus disease (COVID-19) pandemic lockdown in the Netherlands. The CLOFIT dataset contains measures from N = 1910 participants and is broadly representative of the Dutch general population. The dataset represents both sexes, a range of ages including the elderly, different education levels, and ethnic backgrounds. The cohort also includes people with a diverse health status and range of medication use.

## 1. Introduction

In March 2020, the World Health Organization (WHO) officially declared the 2019 coronavirus disease (COVID-19) a pandemic [1]. Due to the rapid spread of the severe acute respiratory syndrome coronavirus 2 (SARS-CoV-2) around the world, and the absence of a vaccine or adequate treatment, the number of hospitalizations and death rates rose quickly. Governments took a variety of preventive measures aiming to reduce the spread of SARS-CoV-2. These measures included “social distancing” (e.g., keeping at least 1.5 m distance from one other, avoiding busy places and crowds), and health and safety measures (e.g., wash and sanitize hands regularly, wear face masks, and stay at home if experiencing COVID-19 symptoms). In addition, in many jurisdictions, partial or total lockdowns were enforced in which people’s time and distance from their domicile were limited (e.g., to one hour per day for a walk) unless for essential work. During these lockdowns, bars and restaurants were usually closed. More stringent lockdowns further closed all schools, daycare, and non-essential shops and businesses, with the exception of essential services such as supermarkets and pharmacies. In several countries, combinations of these measures have been associated with a significant reduction in the number of positive COVID-19 cases and subsequent hospitalizations and death rates.

### 1.1. The Dutch “Intelligent Lockdown”

Dutch “social distancing” measures included keeping 1.5 m distance from other individuals and avoiding busy places and crowds [2]. Health safety measures included advice on not shaking hands, washing and sanitizing hands regularly, coughing and sneezing into the elbow, using paper tissues, and to stay home when experiencing COVID-19 symptoms such as a running nose, fever, or shortness of breath [2]. However, it was not advised to wear facemasks during this first lockdown.

**Figure 1:**
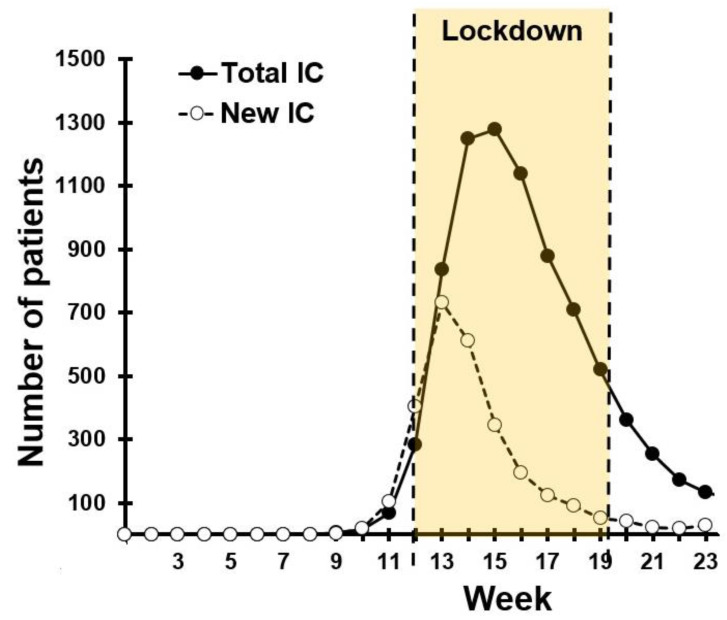
COVID-19 (2019 coronavirus) patients on intensive care units in The Netherlands. Abbreviations: IC = intensive care. Data from reference [3].

Measures to reduce the spread of SARS-CoV-2 infection by the Dutch government were taken in the context of the demand of the pandemic on the limited Dutch intensive care capacity. As is evident from Figure 1, during the first half of March 2020 (i.e., week 11–13, see Figure 1), the demand on intensive care capacity rose exponentially [3]. As a result, a so-called “intelligent lockdown” was enforced from 15 March to 11 May 2020.

**Figure 2:**
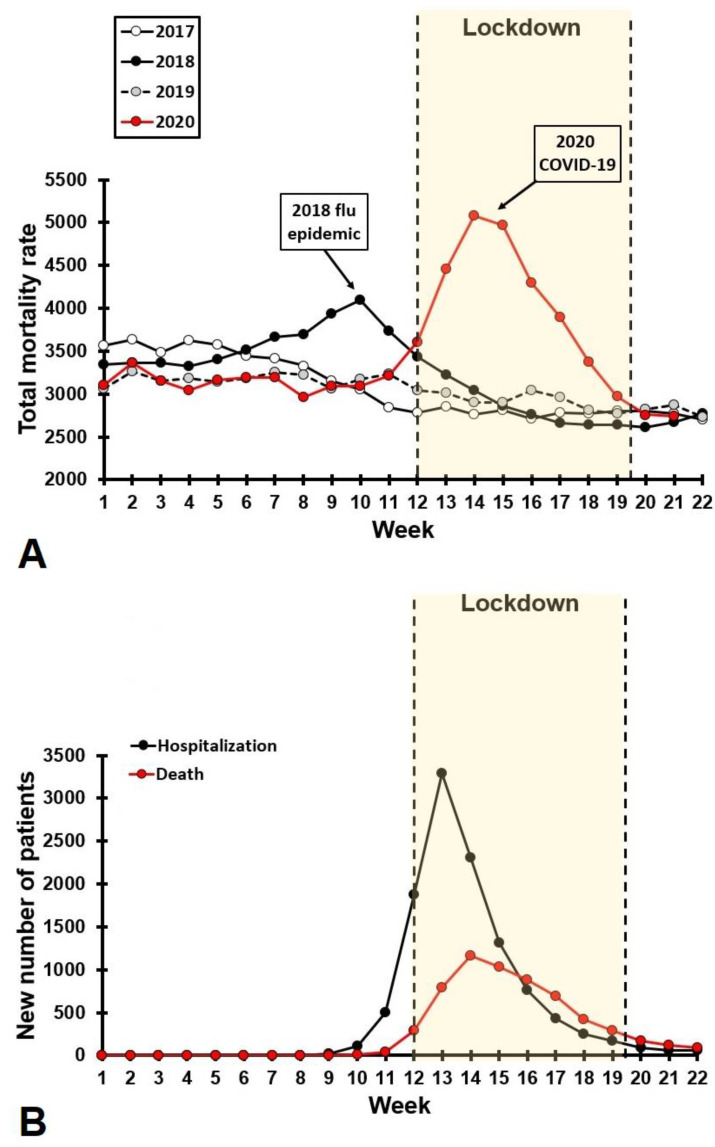
Weekly mortality rates and number of hospitalizations. (**A**) shows the total weekly mortality rate for the Dutch population for 2017–2020. Data were obtained from reference [4]. (**B**) shows the number of confirmed COVID-19 hospitalizations and deaths. Data were obtained from reference [5].

Following the first confirmed COVID-19 case in the Netherlands on 27 February 2020, the first countermeasure was to not shake hands (9 March), followed by the advice to work from home when possible and cancellation of all events with crowds larger than 100 people (12 March). The lockdown measures started on 15 March and included the closure of schools, bars, and restaurants. Except for those with “vital” occupations (e.g., police force, healthcare workers), people were advised to work from home where possible. In addition, people were advised to stay at home, especially if they experienced COVID-19 related health complaints. They could leave home for a short walk or for essential purchases or activities. With the aim of further reducing face-to-face contact, eight days later (23 March 2020), businesses in every sector were closed, with the exception of supermarkets and pharmacies. Leaving home was allowed only for essential activities, and gatherings with more than three people were not permitted. The measures showed to be effective (See Figure 2), and on 11 May 2020, the intelligent lockdown ended and schools and daycare partially reopened. On 1 June, bars, restaurants, theaters and museums were allowed to reopen with a maximum of 30 people per venue, taking into account social distancing and health measures to prevent spreading the virus.

Figure 2A shows the weekly mortality rate for the Dutch population, i.e., the number of confirmed deaths of any cause, recorded by Statistics Netherlands (Centraal Bureau voor de Statistiek, CBS) [4]. Figure 2A illustrates that the initial rise of the mortality rate rapidly reduced during the intelligent lockdown. Mortality rates are usual relatively stable across the year (e.g., see data for 2017 and 2019 in Figure 2A). As a comparator, the increase in mortality rate accompanying the 2018 flu epidemic is included. Weekly mortality rates associated with the first wave of COVID-19 were higher compared to the 2018 flu epidemic, but the duration was shorter. Figure 2B shows the actual number of hospitalizations (intensive care and regular hospital units combined) and number of deaths associated with COVID-19, that have been confirmed through polymerase chain reaction (PCR) testing [5]. Figure 2B shows that after the intelligent lockdown was enforced in the Netherlands, a rapid decrease in reported hospitalizations and death was evident.

### 1.2. Psychological and Health Consequences of a Lockdown

In addition to the expected negative effects on the economy [6], scientific literature shows that social isolation also has a negative impact on psychological and health outcomes [7].

The WHO associates mental health with psychological wellbeing in which an individual is capable of handling their daily life stressors [8]. Pandemics and nationwide lockdowns may result in social isolation which can lead to harmful and long-term negative psychosocial effects, including fear and anxiety of the disease [9]. Studies investigating the psychological and physical health during the 2003 severe acute respiratory syndrome (SARS) outbreak quarantines and lockdowns reported significant increases in negative mood (e.g., increased anxiety and depression), fear of infection, and increased stress [10,11,12]. Other important issues to take into account are confusion, frustration, boredom, anger, and financial uncertainty or unemployment due to the long duration of quarantines [12].

Recent studies from Italy and China revealed significant increases in mental health issues since the start of the COVID-19 outbreak and corresponding lockdowns [13,14,15]. Furthermore, delays in academic activities and employment due to COVID-19 have been associated with poorer mental health outcomes [14]. Parents may also have experienced more stress due to the closure of schools and daycare during lockdowns. In this context, it is important to note that increased stress levels of parents have a direct negative impact on the stress levels of their children [13]. Finally, in addition to social isolation, the excess of (not always correct) information on the Internet and continuous media attention can fuel experiences of negative mood and stress [16].

In the Netherlands, increasing research is devoted to investigating psychological well-being during the COVID-19 lockdown. For example, mixed results were found on sleep quality during lockdown [17] and daily affect and parenting [18] were reported. Social isolation effects due to reduced mobility and work from home have also been described [19]. In addition, studies have investigated health behaviors that may have changed during COVID-19 lockdown, such as smoking cigarettes [20] and eating behavior [21]. However, these studies show that the observed effects that were associated with being in lockdown are not straightforward, and that sociodemographic characteristics and pre-existing conditions have an important impact on the observed study outcomes.

### 1.3. Chronic Stress, Immune Fitness, and Susceptibility to Viral Infection

Increased chronic stress may result in poorer (perceived) immune fitness [22], and low-grade systemic inflammation (i.e., chronic inflammation) may lead to dysregulation of the immune system and in turn negatively affect the body’s response to viruses [23], including SARS-CoV-2.

There is a large body of literature that has demonstrated the negative impact of stress on immune fitness. Acute increased stress levels have been associated with the overexpression of immune biomarkers (e.g., elevated cytokine levels in the blood) [24]. When heightened stress levels persist for a longer time, which could occur during a lockdown or pandemic per se, so-called low-grade inflammation may develop [22]. This is a state of repeated and persistent activation of the immune system and a dysregulated and impaired immune response to for example viruses. Having a poorer immune fitness has been linked to being more susceptible to viral infections. For example, Cohen et al. [25] found that increased psychological stress was associated with a greater likelihood of developing acute infectious respiratory illnesses, including coronavirus type 229E. Another study found that individuals who experienced a chronic stressor in the past year had a higher risk of developing clinical symptoms of the inoculated influenza virus the year thereafter [26]. The risk of developing clinical influenza symptoms was two times higher in participants that experienced stress for more than one month. These studies illustrate a link between stress, immune fitness, and the susceptibility to viral infections. The current study will further explore this relationship in relation to the COVID-19 lockdown in the Netherlands.

### 1.4. Aim of the CLOFIT Study

The purpose of the “Corona lockdown: how fit are you?” survey, referred to as the CLOFIT study, was to investigate the psychosocial and health consequences of the COVID-19 pandemic in the Netherlands. How these consequences relate to immune fitness and reporting COVID-19 related symptom severity was investigated. To this extent, the survey included questions and scales for the period before the lockdown (15 January–14 March 2020) as well as for the intelligent lockdown period (15 March–11 May 2020). The aim of this article is to describe the methodology of the CLOFIT study and a general description of the sample that completed the survey. The outcomes of specific analysis of the data with be presented elsewhere in forthcoming articles.

Several factors may influence how people experience a lockdown, and to what extent circumstances, perceptions, and mood may have an impact on perceived immune fitness and having COVID-19 related symptoms. These issues were covered in the survey and included demographics such as living alone or with others, education level, behaviors such as being active, and health status such as having an underlying disease, body mass index, or the use of medication. The survey evaluated three factors in more detail, namely alcohol consumption, the use of medication, and pain. First, alcohol consumption is an important determinant of health and disease. Anxiety, stress, and loneliness during lockdown are factors that may increase alcohol consumption [27] and subsequent negative effects on immune fitness and health. As such, it was hypothesized that increased alcohol consumption during the lockdown period may aggravate COVID-19-related symptoms. Second, the use of medication during lockdown may have increased (e.g., the use of anxiolytics due to fear of COVID-19) or reduced due to delayed care. It was hypothesized that both effects may become apparent in the data collected though this survey. Third, pain is a frequently reported symptom of viral infections [28]. In addition, changes in pain catastrophizing and associated mood for (chronic) pain unrelated to COVID-19 have been reported during COVID-19 lockdown [29]. Therefore, the presence and severity of pain was assessed in the current survey, including measurements of pain sensitivity and catastrophizing. Finally, we assessed several factors that may improve health such as lifestyle characteristics, including being active and exercise [30,31], and personality characteristics, such as being optimistic and level of mental resilience [32].

**Figure 3:**
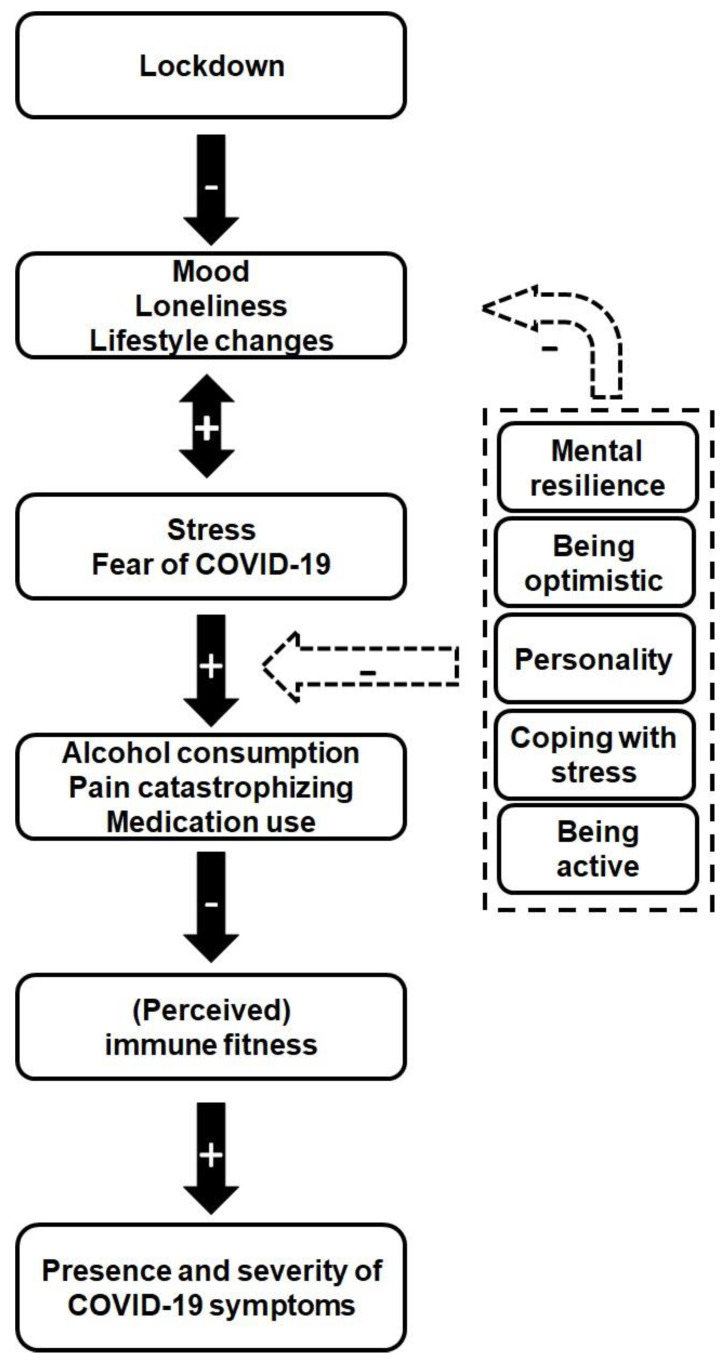
Schematic representation of hypothesized lockdown effects. Note: + sign refers to increase; − sign refers to decrease.

A schematic representation of the hypothesized effects of a lockdown is given in Figure 3. In summary, it is hypothesized that the lockdown had significant negative effects on mood and stress [13,14,15]. These negative effects can however be reduced by having an optimistic personality and being mentally resilient [32], and being active [30,31], illustrated by the negative feedback arrow in Figure 3. The negative mood and stress have a bi-directional relationship (as indicated by the arrow in the figure), and it is hypothesized that, together with fear of COVID-19, they may result in increased alcohol consumption [27], pain catastrophizing [29], and medication use [33]. These factors subsequently are thought to a have negative impact on (perceived) immune fitness [22] and the presence and severity of COVID-19 related symptoms [25].

## 2. Materials and Methods

### 2.1. Design, Population, and Recruitment

The online survey was conducted between the 24 June and the 26 July 2020. The survey was developed and conducted using the online survey platform SurveyMonkey (www.surveymonkey.com), and participants were recruited via Facebook advertisement. In addition, participants from three previous surveys were invited to participate [34,35,36], using an invitation email sent via Survey Monkey. The advertisement asked people to complete a survey entitled “Corona lockdown: how fit are you?”. The advertisement targeted Dutch adults, aged 18 years and older. There were no other inclusion or exclusion criteria, as we aimed to include the general Dutch population. Completion of the survey was voluntary and anonymous. Participants could enter a prize draw, with five vouchers of 100 Euros each, as a reward for completing the survey. Participants could also indicate if they would like to participate in future related research. In that case, they entered their email address, which was stored separately from the data file used for statistical analysis. The Ethics Committee of the Faculty of Social and Behavioral Sciences of Utrecht University granted ethical approval (approval code: FETC17-061), and electronic informed consent was obtained from all participants.

### 2.2. Survey Content and Outcomes

The survey consisted of two parts. Part 1 was the main questionnaire. Part 2 comprised three additional scales (FANTASTIC Lifestyle checklist, Brief mental resilience scale, and the Eysenck personality scale) to obtain more background information of the participants. The scales and questionnaires were completed for different time periods, including 2019, the period before lockdown (15 January–14 March 2020), the lockdown period (15 March–11 May 2020), and the moment of completion of the survey (24 June–26 July 2020). An overview of the assessments is given in Table 1.

#### 2.2.1. Demographics

Demographics included age, sex, weight, height, and city of residence. Body mass index (BMI) was computed. Participants were allocated to one of six groups according to the World Health Organization BMI classification [37]. These include (1) underweight (BMI < 18.5), (2) normal weight (BMI 18.5–24.9), (3) pre-obesity (BMI 25.0–29.9), (4) obesity class I (BMI 30.0–34.9), (5) obesity class II (BMI 35.0–39.9), and (6) obesity class III (BMI ≥ 40).

Ethnicity was reported according to the definitions set by the Statistics Netherlands (Centraal Bureau voor de Statistiek, CBS) [38]. Participants could choose between “Dutch”, “Western migration background”, or “non-Western migration background”. According to the Statistics Netherlands definition [38], a “Western migration background” refers to people descending from European counties (excluding Turkey), North America, Oceania, Indonesia, or Japan. A “non-Western migration background” refers to people from Africa, Latin America, and Asia (excluding Indonesia and Japan, and including Turkey).

#### 2.2.2. Education and Work

Participants could indicate their level of highest education. They could choose between the various specific education types, as listed by Statistics Netherlands [39]. The education types were then recoded into education levels: (1) low, (2) middle, or (3) high education. Low education level comprised VMBO, first three years of HAVO/VWO, or MBO-1. Middle education level comprises completed HAVO/VWO, MBO-2, MBO-3, and MBO-4. High education level comprises HBO or WO.

Participants could indicate whether in 2019 they were employed (owner or employee), unemployed, student, student who also had a job, or retired. If they were employed, they could indicate their job type using the categorization of Statistics Netherlands, adapted for the Dutch population and based on the International Standard Classification of Occupations (ISCO 2008). The Dutch categorization, including average wages for each job category, are listed on the website of Statistics Netherlands [40]. They further stated (a) the number of hours they worked on average per week in 2019 and (b) how many days per week they worked from home or on location (e.g., office). Statistics Netherlands provided information on the average income per job category. In combination with the number of hours per week worked, the monthly income of each participant was estimated.

Absenteeism and presenteeism related to perceived immune fitness were assessed for 2019. Questions were adapted from a recent study examining the cost of workplace hangovers and intoxication to the UK economy [41]. Questions concerned the number of days in 2019 that participants (a) did not work because they experienced reduced immune fitness and (b) did work although they experienced reduced immune fitness. With regard to presenteeism, they could further indicate, in comparison to a regular working day, how well they performed at work on days when they experienced reduced immune fitness. This was done by rating their performance on a scale ranging from 0% (compared to a regular day I achieved nothing/did not work) to 100% (my work was absolutely not influenced by experiencing reduced immune fitness).

With regard to work during the 2020 lockdown period, participants could indicate whether they (a) usually work on location but worked from home during the lockdown period or (b) worked at their usual location (not at home). For their living situation during the lockdown, participants could indicate whether they live alone or with how many other individuals.

#### 2.2.3. Underlying Diseases

Participants could indicate if they had one or more of the following chronic health conditions: none, cardiovascular disease or hypertension, diabetes, liver disease, neurological diseases (e.g., epilepsy, migraine), immune disorders (e.g., rheumatism, Crohn disease), allergy (e.g., hay fever), kidney disease, pulmonary diseases (e.g., chronic obstructive pulmonary disease, asthma), anxiety, depression, sleep disorders, or “other” and report the unlisted disease. The listing of chronic diseases was a selection of those most frequently occurring in adults or the elderly, as listed by the National Institute for Public Health and the Environment (RIVM) [42].

#### 2.2.4. The Use of Medicines

Participants could indicate whether or not they used prescription medicines, including analgesics, antihistaminic drugs, sleep medication, anxiolytics, and antidepressants. The drug categories were chosen as they comprise the most frequently prescribed central nervous system (CNS) drugs. Examples of drug names or indications were given to aid the understanding of items. Participants could indicate whether they did or did not use the medicine. In the case that they used a medicine, they indicated whether or not they used the medication for the first time between 1 March (the date of the first national press conference about the SARS-CoV-2 virus) and the day of completion of the survey. Compared to the 3-month period before the start of the COVID-19 pandemic, participants further indicated whether the amount, frequency, or dose of medicine used was equal, less, or increased during the lockdown. In a similar way, the use of vitamins and probiotics was assessed, as their use may have been increased to strengthen the immune system. Examples of vitamin pills, and probiotic yoghurt drinks were given. Finally, there was a text box provided to comment, give explanations, or add additional over-the-counter (OTC) or prescription medications.

#### 2.2.5. Mood and Quality of Life

Mood was assessed via 1-item scales reflecting some of the subscales of the short version of the Profiles of Mood States (POMS) [43], and included “stress”, “anxiety”, “depression”, “being active”, “fatigue”, and “hostile”. Additional items were “lonely” and “happy”. All items were scored on a scale ranging from 0 (absent) to 10 (extreme). In a similar way, quality of life was assessed. The scale was completed for the period before the lockdown as well as for the lockdown period. Other studies demonstrated that single item visual analog scales are at least equally sensitive and reliable as the full-scale construct assessments of quality of life [44], depression [45], fatigue [46], and alcohol hangover [47]. Single item assessments have been successfully implemented in previous research [48,49,50].

#### 2.2.6. Fear of COVID-19 (FCV-19S)

The Fear of COVID-19 Scale (FCV-19S) is a seven-item scale, assessing feelings of fear related to COVID-19 [51]. An example of the items is “When I am watching news and stories about corona on social media, I become nervous or anxious”. In the original FCV-19S, each item can be answered on a Likert scale ranging from 1 (strongly disagree) to 5 (strongly agree). The higher the sum score of the items, the higher the fear of COVID-19. The FCV-19S has a Cronbach’s alpha of 0.82, and sum scores correlated significantly with depression and anxiety scores of the Hospital Anxiety and Depression Scale (r = 0.425 and r = 0.511, respectively), and the perceived infectability and germ aversion subscales of the Perceived Vulnerability to Disease Scale (r = 0.483 and r = 0.459, respectively) [51]. In the current version of the FCV-19S, the items were translated into Dutch by the authors. We modified the scoring range into −2 (strongly disagree) to 2 (strongly agree), around a midpoint of 0 (neutral). This scoring rage may be easier to interpret, including possible improvement/aggravation in assessments at different timepoints and their relation to the normal “neutral” state. Further, participants were allocated to one of two groups, including “fear of COVID-19” (sum score greater than 0) or “no fear of COVID-19” (sum score ≤ 0). The FCV-19S was completed for the lockdown period and for comparison reasons also for the moment of completion of the survey.

#### 2.2.7. Immune Status Questionnaire (ISQ)

To assess the past year’s immune fitness (i.e., the year 2019), the Immune Status Questionnaire (ISQ) was completed [52]. The ISQ consists of seven items, including “common cold”, “diarrhea”, “sudden high fever”, “headache”, “muscle and joint pain”, “skin problems (e.g., acne and eczema)”, and “coughing”. The items are scored on a 5-point Likert scale stating how often participants experienced these complaints during the past year, including “never”, “sometimes”, “regularly”, “often”, and “(almost) always”. The overall ISQ score ranges from 0 (poor) to 10 (excellent), with higher scores indicating a better perceived immune fitness. Cronbach’s alpha was 0.632 with a test-retest reliability of 0.80 [51].

#### 2.2.8. Perceived Immune Fitness

To directly assess perceived immune fitness at specific time points, immune fitness was also assessed using a 1-item scale ranging from 0 (poor) to 10 (excellent), with higher scores indicating a better perceived immune fitness [52,53]. Perceived immune fitness was rated for the year 2019, for the period before the lockdown, the lockdown period, and after the lockdown (i.e., the moment of completion of the survey).

#### 2.2.9. The Jackson Symptoms Scale—Common Cold

The Jackson Symptoms Scale [54] was developed to assess common cold symptoms and comprises eight items, including sneezing, running nose, sore throat, cough, headache, nasal congestion, chills, and malaise/feeling sick. The severity of each of the eight items could be rated as none (0), mild (1), moderate (2), or severe (3). Individual item scores are summed to create a total symptom score, with a possible range from 0 (no complaints) to 24 (severe complaints). For the current study, the authors translated the scale into Dutch. The Jackson Symptoms Scale was completed for the period before the lockdown and during the lockdown period.

#### 2.2.10. COVID-19 Symptoms Scale (C-19SS)

The Jackson Symptoms scale [54] was modified to develop the COVID-19 Symptoms Scale (C-19SS). For the COVID-19 Symptoms Scale, five items of the Jackson Symptom Scale (sneezing, running nose, sore throat, cough, and malaise/feeling sick) were retained, and four COVID-19 specific items were added, comprising high temperature (up to 38 Celsius), fever (38 Celsius and higher), shortness of breath, and chest pain. The severity of each of the nine items could be rated as none (0), mild (1), moderate (2), or severe (3). Individual item scores are summed to create a total symptom severity score, with a possible range from 0 (no complaints) to 27 (severe complaints). In addition, the presence of COVID-19 symptoms was calculated by counting the number of symptoms with a score > 0. The C-19SS was completed for the period before the lockdown and the lockdown period.

#### 2.2.11. COVID-19 Test

Participants could indicate whether or not they had been tested for COVID-19. If they had tested positive, they could indicate whether they (a) had been hospitalized, (b) had been sick at home, or (c) did not feel very sick. If they had not been tested for COVID-19, they could indicate whether or not they thought they had been previously infected with SARS-CoV-2 and suffered from COVID-19.

#### 2.2.12. Alcohol Consumption

Questions about alcohol consumption were answered for the period before the lockdown and during the lockdown period. Questions from the Quick Drinking Screen were modified for the purpose of this study [55]. For both periods, participants reported the number of alcoholic drinks they consumed on average per week, and the number of days per week they consumed alcohol. Guidance was provided regarding drinking sizes and how to convert these into units of alcohol. With regard to the heaviest drinking occasion within each of the two periods, the number of alcoholic drinks consumed as well as the duration of drinking (h) was reported. The estimated blood alcohol concentration (BAC) for this occasion was computed using an adapted Widmark equation [56], taking into account sex and body weight. Subjective intoxication (drunkenness) for these occasions was rated on an 11-point scale ranging from 0 (totally not) to 10 (extremely drunk) [57]. Using a similar scale, next day hangover severity was assessed, with a range from 0 (no hangover) to 10 (extremely severe) [44]. Finally, participants reported how many hangovers they had experienced before and during lockdown.

#### 2.2.13. Pain Sensitivity Questionnaire (PSQ)

Sensitivity to pain was assessed with the Pain Sensitivity Questionnaire (PSQ) [58,59]. Items of the PSQ address different thermal, chemical, and mechanical pain modalities and body sites. For this study, the shortened 10-item version of the PSQ was used [60], which was translated into Dutch by the authors. For each item, pain intensity was rated on a scale of 0 (“no pain”) to 10 (“most intense pain imaginable”). The total PSQ score is calculated as the average rating of the 10 items. Higher scores indicate a greater sensitivity to pain. The PSQ was completed for the moment the survey was held. Cronbach’s alpha of the PSQ is 0.91 [60].

#### 2.2.14. Pain Catastrophizing Scale (PCS)

Pain catastrophizing has been broadly defined as an exaggerated negative orientation towards actual or anticipated pain experiences and has been described as the tendency to recall pain experiences in more exaggerated terms, to feel helpless and ruminate over painful events [61]. The shortened Pain Catastrophizing Scale (PCQ) was used in the current study [62,63]. The scale comprises three questions which can be scored from 1 “not at all” to 5 “always”, addressing the modalities rumination, magnification, and helplessness. The items are considered individually and a sum score is calculated to reflect overall pain catastrophizing. Higher scores correspond to greater pain catastrophizing. Overall PCQ scores ≥ 8 are regarded clinically significant. The PCS was completed for the moment the survey was held. Cronbach’s alpha of the shortened PCS is 0.892 [62].

#### 2.2.15. Graded Chronic Pain Scale—Revised (GCPS-R)

The Graded Chronic Pain Scale (GCPS) includes questions on the intensity of the pain, as well as the impact pain may have on the enjoyment of life, general activity, and work [64]. For this survey, the revised 6-item version of the scale (GCPS-R) was used [65] which was translated into Dutch by the authors. Item 1 (“How often did you have pain?”) and item 2 (“How often did pain limit your life or work activities?”) could be answered by “never”, “some days”, “most days”, or “every day”. Item 3 (“What best describes your pain, on average?”), was rated on an 11-point scale ranging from 0 (no pain) to 10 (pain as bad as you can imagine). Item 4 (“What number describes best how pain has interfered with your enjoyment of life?”) and item 5 (“What number describes best how pain has interfered with your general activity?”) are rated on an 11-point scale ranging from 0 (does not interfere) to 10 (completely interferes). Finally, item 6 (“Are you not working or unable to work due to a pain or a pain condition?”) could be answered by “yes” or “no”. The six items are scored and evaluated individually. In addition, using the scoring method by Von Korff et al. [65], participants can be allocated to one of four categories, including “chronic pain absent”, “mild chronic pain”, “bothersome chronic pain”, or “high impact chronic pain”. The GCPS-R was completed for 2019 and the lockdown period.

#### 2.2.16. Use of Pain Medication

In addition to information on medication use discussed in Section 2.2.4, additional information was collected on the use of pain medication. Participants could indicate whether or not they used one or more pain medications, including amitriptyline, aspirin (acetylsalicylic acid), carbamazepine (tegretol), celecoxib (celebrex), codeine, diclofenac (cataflam/voltaren gel of tablet), etoricoxib, fentanyl (abstral/recivit/durogesic), gapapentin (neurontin), ibuprofen (advil/brufen), meloxicam, morfine (oramorph), naproxen (aleve), nortriptyline (nortrilen), oxycodon (oxycontin/oxynorm), paracetamol, pregabaline (lyrica), tramadol (tramal), triptans (sumatriptan, rizatriptan, etc.), or other (open-ended question). The selection of medicines for inclusion in the survey was based on the current prescription practices of pain medication in The Netherlands, listed in the “Farmacotherapeutisch Kompas” [66], and according to the pain treatment guidelines of the Dutch College of General Practitioners [67]. The question was completed for 2019 and for the lockdown period.

#### 2.2.17. The FANTASTIC Lifestyle Checklist

The original FANTASTIC Lifestyle Checklist comprises 25 questions assessing different domains, including (F) support of family and friends, (A) physical activity level, (N) nutrition, (T) tobacco and toxins, (A) alcohol, (S) sleep, seatbelts, stress, safe sex (T) type of personality, (I) insight, and (C) career [68,69]. Each item has 5 answering possibilities corresponding to a score ranging from 0 to 4 [70]. For the current survey, the checklist was translated into Dutch by the authors. However, a modified version of the FANTASTIC Lifestyle Checklist was used [71]. The alcohol consumption questions were not included as this topic was covered elsewhere in the survey (see Section 2.2.12). In addition, the original question on drug use (domain T) had two answering possibilities (sometimes or never), which was changed into five answering possibilities, including almost daily (0), fairly often (1), only occasionally (2), almost never (3), or never (4). Total scores were computed for the domains (F) support of family and friends, (A) physical activity level, (N) nutrition, (T) tobacco and toxins, and (C) career. Higher scores on items or scales suggest a better or healthier lifestyle. For domain S (“sleep, seatbelts, stress, safe sex”), separate scores were computed for sleep (“I sleep well and feel rested”), safety (items “I use seatbelts”, and “I practice safe sex” combined), and coping (items “I am able to cope with the stresses in my life”, and “I relax and enjoy leisure time” combined). Items of the domains (T) type of personality (“I seem to be in a hurry”, and “I feel angry or hostile”), and (I) insight (“I am a positive or optimistic thinker”, “I feel tense or uptight”, and “I feel sad or depressed”) were considered individually. The modified FANTASTIC Lifestyle Checklist was completed for the period before and during the lockdown.

#### 2.2.18. Mental Resilience

Mental resilience was assessed using the Brief Resilience Scale (BRS) [72]. The BRS consists of 6 items and measures the ability to recover from stress, i.e., to bounce back. BRS items are can be endorsed on a 5-point Likert scale ranging from 1 (“strongly disagree”) to 5 (“strongly agree”). A higher BRS score indicates higher mental resilience, i.e., faster recovery from stress. Items 1, 3, and 5 are positively worded, and items 2, 4, and 6 are negatively worded, and therefore reverse coding is applied to items 2, 4, and 6. The BRS has a Cronbach’s alpha ranging from 0.80 to 0.91 [72]. Previous research using a Dutch version of the BRS [53] showed that BRS scores significantly correlated with personality characteristics, psychological coping strategies, perceived immune fitness, and health outcomes [53,72]. The BRS was completed for the moment the survey was held.

#### 2.2.19. Personality

Personality traits were assessed using the Dutch version of the Eysenck Personality Questionnaire (EPQ-RSS) [73,74]. This 48-item questionnaire consists of 4 subscales, assessing psychoticism, extraversion, neuroticism, and socialization. Each subscale consists of 12 items, which can be answered with “yes” or “no”. Scores are 0 or 1, with reversed scoring applied to selected items [69]. Subscale scores range from 0 to 12, with higher scores implying that participants score higher on the personality trait. Cronbach’s alpha for the Dutch EPQ-RSS scales were 0.35–0.521 for psychoticism, 0.81–0.84 for neuroticism, 0.72–0.84 for extraversion, and 0.69–0.76 for socialization [74]. The EPQ-RSS was completed for the moment the survey was held.

#### 2.2.20. Concluding Questions and Remarks

At the end of the survey, participants were advised to consult their physician if they feel they are experiencing COVID-19 symptoms. They were referred to the website of the Dutch RIVM (https://www.rijksoverheid.nl/onderwerpen/coronavirus-covid-19 accessed on 15 October 2020) for further information on COVID-19. For more information on alcohol or drug use, they were referred to the Trimbos Institute (www.trimbos.nl accessed on 15 October 2020).

### 2.3. Data Handling and Statistical Analysis

Data were collected via Survey Monkey (www.surveymonkey.com accessed on 15 October 2020) and downloaded in Excel format. The data were recoded according to instructions from original scales and transferred to SPSS (IBM Corp. Released 2013. IBM SPSS Statistics for Windows, Version 25.0. Armonk, NY, USA: IBM Corp.). For the description of the sample, mean and standard deviation (SD) of the total scale scores and subscale scores were computed, and the percentage of subjects that fall in the descriptive categories such as education level and sex.

## 3. Results

N = 2251 participants entered the starting page survey, which provided a brief background of the purpose of the survey, and an informed consent button to start the survey. Of these, N = 83 did not provide informed consent and N = 242 participants provided informed consent but did not start the survey.

Data were checked case by case to verify that it was reliable. Participants with unreliable or incompatible data were excluded. Unreliable data was identified by evaluating outliers for individual variables. For example, participants reporting a height of less than 1 m or a weight below 40 kg were considered unreliable. Incompatible data was identified by comparing answers to different questions and observing that these contradict each other. For example, (a) participants indicating having all chronic diseases listed, but at the same time report using no medicines, or (b) participants indicating drinking alcohol 1 day per week, but reporting 4 hangovers per week. Data from N = 20 participants were judged unreliable or incompatible and these participants were excluded from the dataset. Data from the other N = 1910 participants formed the final CLOFIT study dataset. N = 571 participants (29.9%) gave informed consent to complete part 2 of the survey. N = 598 subjects (31.3%) indicated that they may be contacted to participate in future to participate in follow-up research. As not all of the participants completed part 1 and 2 of the survey, and some questions were not relevant to all participants (e.g., questions about work for participants that are student of unemployed), the number of participants per assessment within the survey varies (see Table 2).

**Figure 4:**
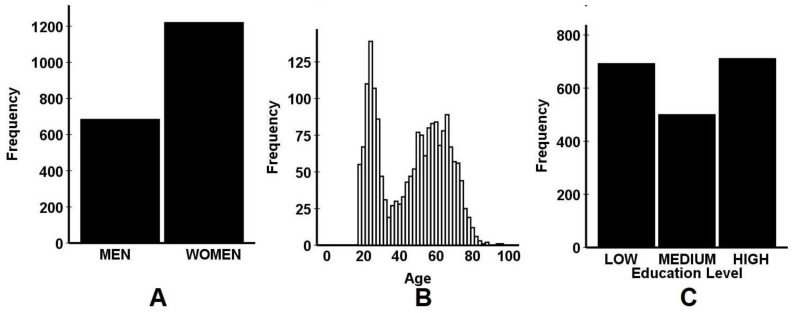
Main demographics of the CLOFIT database. The sample distribution is presented according to sex (**A**), Age (**B**), and education level (**C**).

Table 3 and Figure 4 give an overview of the main demographic variables of the sample. More females than males participated in the survey (see Figure 4A), although both groups have sufficient sample size to evaluate potential sex differences. Moreover, different age groups are sufficiently presented (see Figure 4B). Although relatively few participants were included in the 30–40 years old age range, the data allow comparisons between young adults (18–30 years old), adults (30–64 years old), and the elderly (65 years and older). Although not in line with nationally representative samples, the relative equal distribution of participants across educational levels (i.e., low, medium, versus high) also results in sufficient sample sizes for statistical comparisons (see Figure 4C).

A substantial number of participants reported having underlying diseases (64.8%). This percentage is higher than observed in the total Dutch population. One could speculate that individuals at risk for COVID-19 infection such as elderly and those with underlying diseases are more interested in completing a survey on COVID-19 and may therefore be overrepresented in the CLOFIT sample. In addition, women are sightly overrepresented (64%), compared to the fairly equal male/female distribution in the Dutch population (49.7% versus 53.3%, respectively). In previous online surveys, we also noted that, compared to men, women were more frequently attracted to complete surveys related to health issues [35,75].

Finally, the mean BMI of the CLOFIT sample of 26.5 kg/m^2^ is relatively high and suggest an overweight population. Inspection of the data revealed that 55 participants had a BMI > 40, and statistical analysis determined these as outliers. The high BMI was most commonly caused by a combination of a high body weight and a short height and the fact that 85.7% of these individuals reported having underlying diseases such as cardiovascular disease or diabetes. Without the 55 participants with a BMI ≥ 40, the mean (SD) BMI of the CLOFIT sample is 25.9 (4.8).

## 4. Discussion

The online survey of the CLOFIT study provided a comprehensive dataset to investigate the psychosocial and health consequences of the intelligent lockdown during the COVID-19 pandemic in the Netherlands and its relationship with (perceived) immune fitness and reported COVID-19 related symptoms.

The structure of the sample that completed the survey allows comparisons between age groups and to evaluate potential sex differences. It also comprises sufficient sample size to investigate the possible impact of having underlying diseases on other study outcomes. Finally, sufficiently powered subsamples reported on alcohol consumption, pain, and medication use, allowing analysis of these correlates of COVID-19 lockdown and their relationship with perceived immune fitness and reporting COVID-19 symptoms in more detail.

### 4.1. Limitations

There are some limitations to the CLOFIT study that should be addressed. First, all data were self-reported. Therefore, recall bias may have played a role. In addition, at the time of collecting the data, participants may to some extent have even idealized their emotional state prior to the lockdown. It is unclear to what extent this may have influenced the outcome of the study. Second, the sample size is relatively small. From the responses were received on the Facebook account corresponding to the survey invitation, it appeared that a substantial number of people were overwhelmed and bored with the COVID-19 topic and were therefore not willing to complete the survey. Third, the 30–50 years old age group is relatively underrepresented in the final sample (see Figure 4B). Nevertheless, all age groups, education levels, and both sexes are sufficiently represented in the final CLOFIT sample. However, the planned comparisons between job categories must be reconsidered, as several of the samples of job categories are too small.

Fourth, another issue to discuss is the development and use of the COVID-19 Symptoms Scale (C-19SS) in future analysis. At the time of designing the CLOFIT study (the first quarter of 2020), relevant known symptoms were included that were listed by the National Institute for Public Health and the Environment (RIVM) [42]. However, according to current literature, the symptoms included in the C-19SS are not exhaustive, as other symptoms were added more recently. For example, in April, the US Centre for Disease Control and Prevention (CDC) listed only six COVID-19 symptoms, including “loss of taste or smell”, “chills”, “shaking with chills”, “muscle pain or aches”, “sore throat”, and “headache”. On 13 July 2020, the CDC added “nausea”, “diarrhea”, and “runny nose” to their listing of symptoms. Literature shows that symptoms such as “loss of smell and taste”, reported by 5–95% of COVID-19 patients [76], and “diarrhea”, reported by 13.5% of COVID-19 patients [77]. These symptoms were not included in the C-19SS. The impact of these symptoms on overall COVID-19 symptom severity is unknown and there is great variability in the number of reported cases. The latter is probably due to methodological differences to assess loss of taste and smell between the studies and because of difference samples that were investigated (e.g., young adults with minimal complaints versus hospitalized elderly). Notably, also among the general population of US (not infected with SARS-CoV-2) loss of taste and smell is reported by 13.5% and 17.3%, respectively [78]. Therefore, the CDC refers to “new” loss of taste and smell in their current symptom listing. Another serious but infrequently reported symptom “loss of speech or movement” was also not included in the C-19SS. To date, these symptoms are not listed by RIVM as characteristic COVID-19 symptoms suggesting that individuals should apply for testing for SARS-CoV-2 infection [42]. Finally, it should be mentioned that CLOFIT data allows for correlational analysis, but that causality cannot be inferred from the current study design.

### 4.2. Future Research Directions

It is intended to contact participants who agreed to invite them for follow up assessments. These will comprise surveys assessing health correlates regarding the period after the first COVID-19 wave. The continuing increase of knowledge on the development and pathology of COVID-19 will help further improve symptom assessment scales. In future research, the omitted symptoms will be included in a revised version of the C-19SS. Notwithstanding the omitted symptoms, given that the most commonly reported COVID-19 symptoms are included in the C-19SS, we believe the currently used version of the C-19SS provides a valid and reliable measure to characterize the presence and severity of experiencing COVID-19 symptoms. In addition, via collection of saliva or blood samples it is aimed to assess biomarkers of immune fitness in a subsample of participants, to serve as objective measures next to the assessments of perceived immune fitness. The analysis of the current data, including the answers to open questions, will, together with the development of the COVID-19 pandemic, determine the exact research aims of future research. Most likely, data will be collected using online surveys. It is not intended to use digital technologies such as mobile phone applications to conduct real-time assessments [79,80], nor have we planned to conduct any interventions. However, the potential long-term consequences of lockdown and the pandemic will be assessed in follow-up research, including mood and health correlates, and how these might have been affected by lifestyle factors and behaviors.

## 5. Conclusions

The online survey of the CLOFIT study has provided a comprehensive dataset to investigate the psychosocial and health consequences of the COVID-19 pandemic lockdown in the Netherlands and its relationship with (perceived) immune fitness and reported COVID-19 related symptoms.

## Figures and Tables

**Table 1:** Assessments of the “Corona lockdown: how fit are you?” (CLOFIT) survey.

Questionnaire/Scale	2019	Before Lockdown	During Lockdown	Moment of Survey Completion
Demographics	-	-	-	√
Education	-	-	-	√
Employment status	√	-	√	-
Work and immune fitness	√	-	-	-
Work and pain	√	-	-	-
Work and alcohol hangover	√	-	-	-
Underlying diseases	-	-	-	√
The use of medicines	-	√	√	-
Mood and quality of life	-	√	√	-
Fear of COVID-19 (FCV-19S)	-	-	√	√
Immune Status Questionnaire (ISQ)	√	-	-	-
Perceived immune fitness	√	√	√	√
The Jackson Symptoms scale—common cold	-	√	√	-
COVID-19 Symptoms Scale (C-19SS)	-	√	√	-
COVID-19 test	-	-	-	√
Alcohol consumption	-	√	√	
Pain Sensitivity Questionnaire (PSQ)	-	-	-	√
Pain catastrophizing scale (PCS)	-	-	-	√
Graded Chronic Pain Scale—Revised (GCPS-R)	-	-	-	√
Use of pain medication	√	-	√	-
The FANTASTIC Lifestyle Checklist	-	√	√	-
Mental resilience	-	-	-	√
Personality	-	-	-	√

Note: √ = assessed, - = not assessed.

**Table 2:** Number of participants that completed the survey components.

Questionnaire/Scale	Description in Section	Number of Participants
*Part 1 of the survey*		
Demographics	2.2.1	1910
Education	2.2.2	1910
Employment status	2.2.2	907
Work and immune fitness	2.2.2	495
Work and pain	2.2.2	238
Work and alcohol hangover	2.2.2	364
Underlying diseases	2.2.3	1378
The use of medicines	2.2.4	1415
Mood and quality of life	2.2.5	1415
Fear of COVID-19 (FCV-19S)	2.2.6	1020
Immune Status Questionnaire (ISQ)	2.2.7	1408
Perceived immune fitness	2.2.8	1020
The Jackson Symptoms scale—common cold	2.2.9	1020
COVID-19 Symptoms Scale (C-19SS)	2.2.10	1020
COVID-19 test	2.2.11	1009
Alcohol consumption	2.2.12	761
Pain Sensitivity Questionnaire (PSQ)	2.2.13	910
Pain catastrophizing scale (PCS)	2.2.14	910
Graded Chronic Pain Scale—Revised (GCPS-R)	2.2.15	495
Use of pain medication	2.2.16	495
*Part 2 of the survey*		
The FANTASTIC Lifestyle Checklist	2.2.17	514
Mental resilience	2.2.18	511
Personality	2.2.19	505

**Table 3:** Demographics.

Variable	Study Outcome
Male/Female	687 (36.0%)/1223 (64.0%)
Age (years)	46.3 (18.5)
Weight (kg)	79.2 (18.6)
Height (m)	1.73 (0.1)
BMI (kg/m^2^)	26.5 (5.8)
Ethnicity—Dutch	1796 (94.0%)
—Migration background	114 (6.0%)
Educational level—Low	694 (36.4%)
—Middle	502 (26.3%)
—High	713 (37.3%)
Employment status—Unemployed	146 (16.1%)
—Employer/employee	262 (39.1%)
—Student	86 (9.5%)
—Student with parttime job	153 (16.9%)
—Retired	160 (17.6%)
Underlying disease—Yes	893 (64.8%)
—No	485 (35.2%)

Results for age, weight, height, and BMI are presented as mean (SD); other variables as number (%). Abbreviation: BMI = body mass index, SD = standard deviation.

## Data Availability

The survey and data are available upon request from the corresponding author.
